# A giant hemolymphangioma of the pancreas in a 20-year-old girl: a report of one case and review of the literature

**DOI:** 10.1186/1477-7819-7-31

**Published:** 2009-03-18

**Authors:** Li-Feng Sun, Hui-Lin Ye, Qi-Yan Zhou, Ke-Feng Ding, Pei-Lin Qiu, Yong-Chuan Deng, Shu-Zhan Zhang, Shu Zheng

**Affiliations:** 1 Department of Surgical Oncology, the Second Affiliated Hospital, College of Medicine, Zhejinag University, Hangzhou Zhejiang 310009, PR China; 2 People's Hospital of Songyang, Songyang, Zhejiang 323400, PR China; 3 Zhejiang Qingchun Hospital, Hangzhou, Zhejiang 310013, PR China

## Abstract

**Background:**

Hemolymphangioma of the pancreas is a very rare benign tumor. There were only six reports of this disease until December 2008. Herein, we report a case of giant hemolymphangioma of the pancreas in a 20-year-old girl.

**Case presentation:**

We describe a 20-year-old girl who presented with a mass in abdominal cavity and epigastric discomfort about a week. Physical examination showed a great abdominal mass. Abdominal computed tomography showed extrinsic duodenal compression due to a large retroperitoneal tumor possibly arising from pancreas. The tumor enucleation was performed and a diagnosis of hemolymphangioma of the pancreas was made. The patient had a complication of chylous leakage, which was successfully managed. The patient is alive and well, after 26 months of follow-up, with no complaints or recurrence.

**Conclusion:**

From this case and literature, we can conclude that hemolymphangioma of the pancreas in adult is a rare benign tumor, and accurate diagnosis can not be preoperatively established. Tumor resection should be performed whenever possible. The risk of recurrence seems very low.

## Background

Hemolymphangioma of the pancreas is a rare disease and basically benign cystic tumor. There were only six reports of this tumor of the pancreas until December 2008 (PubMed) [1-6]. Cystic tumors of the pancreas account for approximately 10% to 15% of cystic lesions of the pancreas. Vascular tumors of the pancreas are cystic tumors accounting for 0.1% of all pancreatic tumors[7]. Major symptoms in this hemolymphangioma are a mass in abdominal cavity and epigastric discomfort associated with the enlarged tumor. We present a large hemolymphangioma of the pancreas in a 20-year-old girl with a review of the literature.

## Case presentation

**Figure 1:**
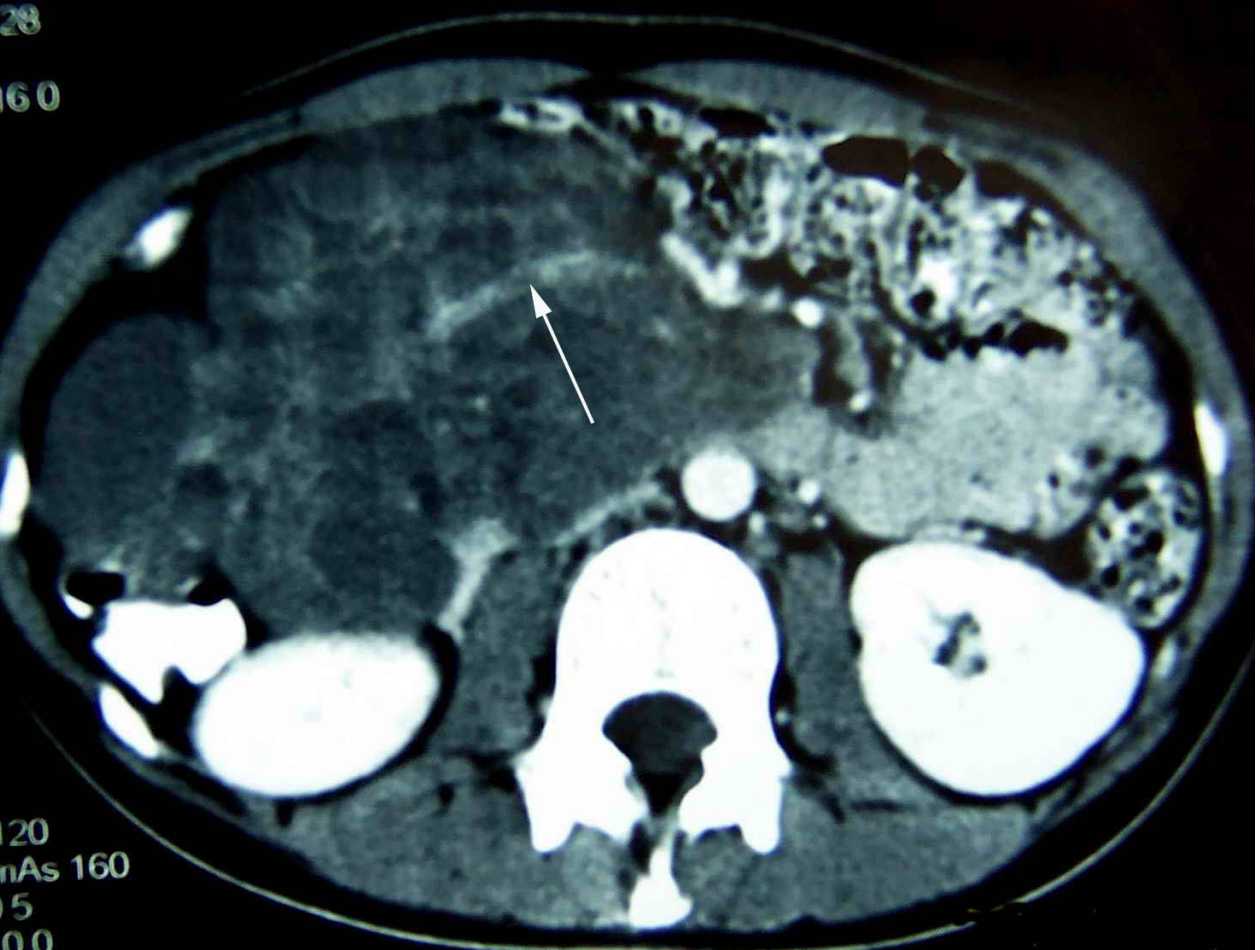
**Abdominal Computed tomography demonstrating a large tumour with partial blood flow(arrow) in abdominal cavity**.

The patient was a 20-year-old girl who complained of a mass in abdominal cavity and epigastric discomfort about a week. She was a college student. On admission she was well, not vomit, stomachache and icteric. Physical examination showed a large abdominal mass. Abdominal computed tomography showed extrinsic duodenal compression due to a large retroperitoneal tumor possibly arising from pancreas, which had polycystic structure and partial blood flow (Figure 1). No abnormalities were revealed in laboratory data including tumor markers such as CEA and CA19-9. At laparotomy there was a black polycystic, retroperitoneal tumor extending from coeliac axis to the origin of the head of pancreas. The tumor infiltrated the transverse mesocolon, greater omentum, and tightly adhered to duodenum and superior mesenteric artery. Along the surface of the duodenum and pancreas, tumor (including partial transverse mesocolon and greater omentum) excision was performed. Pancreatoduodenectomy was not performed.

**Figure 2:**
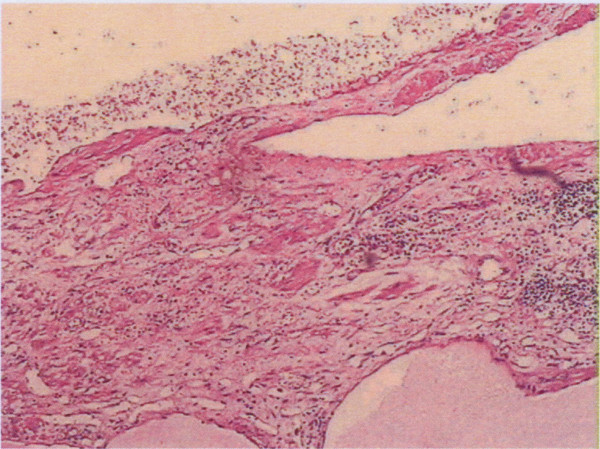
**Low-power review showing a soft tissue mass consists of lymphatic and blood vessels with polycystic spaces (H & E ×100)**.

Macroscopically, the mass measured 18 × 16 × 12.5 cm. It was nodular, and soft in consistency. The tumors were multiloculated cystic masses full of bloody fluid. Microscopically(Figure 2), the tumor showed a soft tissue mass consisted of lymphatic and blood vessels with polycystic spaces. There was infiltration of the stroma by lymphocytes. The definitive histological diagnosis was hemolymphangioma of the pancreas.

After operation, the patient had a complication of chylous leakage, which was successfully managed. She was cured and dischaged after 20 days after surgery. After 26 months of follow-up by computed tomography and Ultrasonography, there was no complaints or recurrence.

## Discussion

Intra-abdominal hemolymphangioma is very rare; rarer still is the involvement of pancreas. On a review of published work (online PubMed search) till December 2008 (Pubmed), we found only 6 case reports [1-6]. Their characteristics were showed below (Table 1). This tumor is considered a congenital malformation of the vascular system. The formation of this tumor may be explained by obstruction of the venolymphatic communication between dysembrioplastic vascular tissue and the systemic circulation[5]. These lesions may arise from the pancreatic parenchyma[5].

**Table 1:** Characteristics of six patients with hemolymphangioma of the pancreas[5]

Case No.	Age(y)	sex	Site of pancreas	Size/weight	Treatment	Prognosis
1	68	F	head	-/1450 g	PD	No recurrence
2	66	F	head	-/1500 g	PD+PG	No recurrence
3	31	F	Body/tail	14 cm/-	BTP	No recurrence
4	67	F	head	15 cm/-	PD	No recurrence
5[5]	53	F	head	4*3 cm/-	PD	No recurrence
6[6]	53	M	head	>6 cm/-	PD	No recurrence

PD, pancreatoduodenectomy; PG, partial gastrectomy; BTP, body and tail pancreatectomy.

Hemolymphangioma of pancreas are usually large lesions with a diameter of larger than 10 cm, and the commonest site is the head of pancreas. Generally, they are large masses with thin wall having multiple thin septa with varying size cystic cavities containing fluid similar to hemorrhagic and rarely of clear lymphatic nature. Microscopically, the tumor consists of abnormal lymphatic and blood vessels with polycystic spaces. These cysts have connective septa covered by endothelium.

This tumor may be asymptomatic for a long time. Abdominal pain and awareness of abdominal mass are the most common symptoms. Other infrequent symptoms such as vomiting and nausea are caused by occupied tumor. This tumor is commonly a benign disease and has no invasion ability. But in the 6th case reported by a Japanese group[6], the chief complaint was severe anemia caused by duodenal bleeding because the hemolymphangioma of the pancreas invaded to the duodenum. This symptom is extremely rare. In our case, the chief symptoms were a giant mass in abdominal cavity and epigastric discomfort. At laparotomy tumor infiltrated the transverse mesocolon and greater omentum, and tightly adhered to the duodenum and superior mesenteric artery. Generally, this disease is benign, but it is possible that this tumor invaded other organs like our case and Japanese case.

The clinical diagnosis of hemolymphangioma of pancreas is not often due to its rarity and the absence of clinical expression. Laboratory tests are frequently normal although the case reported by Banchini [4] had a slight increase in alkaline phosphatase and gamma-glutamyl transferase. Serum carcinoembryonic antigen (CEA) and CA19-9 are within normal limits. Imaging techniques such as ultrasonography, Abdominal Computed tomography, and magnetic resonance imaging may be used to assess, make a clinical diagnosis and for follow-up. The impossibility to preoperatively define the histological type of the tumor explains the difficulties to reach a correct differential diagnosis. Clinical differential diagnoses includes pseudocyst, lymphangioma, serous from mucinous tumors, sarcoma, enteric duplication cyst, and cystic tumor not otherwise specified. The final diagnosis is based on a combination of clinical, radiological, and histopathological findings.

Surgery including local resection of this tumor is a definitive modality. Two operative attitudes are possible: the tumoral enucleation and the partial pancreatectomies. Hemolymphangioma of the pancreas is commonly a benign disease and has no invasion ability. Local resection is necessary. But in the Japanese case, pancreatoduodenectomy was performed because the tumor invaded to the duodenum to cause the duodenal bleeding. In addition, pancreatoduodenoctomy is performed for suspicion of malignancy. All cases in the literature had good prognosis as did our case. The risk of recurrence or metastasis seems very low, but careful follow-up is necessary.

Herein, we reported a case of hemolymphangioma of the pancreas head with a large size in a 20-year-old girl. The wide infiltration and adherence of adjacent organs and tissues was an important feature of the present case.

## Conclusion

From this case and literature, we can conclude that hemolymphangioma of the pancreas in adult is a rare benign tumor, and accurate diagnosis can not be preoperatively established. Tumor resection should be performed whenever possible. The risk of recurrence seems very low. Despite its low frequency, this disease should be considered when a multiloculated cystic masses in abdominal cavity is seen.

## Consent

Written consent was obtained from the patient or their relative for publication of study.

## Competing interests

The authors declare that they have no competing interests.

## Authors' contributions

LFS and HLY designed the study, performed picture acquisition and drafted part of the manuscript. PLQ and KFD performed the surgery, carried out data acquisition and drafted part of the manuscript. All authors participated in the editing and have read and approved the final manuscript.
